# Comprehensive geriatric assessment for predicting postoperative delirium in oral and maxillofacial surgery: a prospective cohort study

**DOI:** 10.1038/s41598-024-78940-z

**Published:** 2024-11-11

**Authors:** Eman Alhammadi, Julian Max Kuhlmann, Majeed Rana, Helmut Frohnhofen, Henriette Louise Moellmann, Nonauthor Collaborator (Data Collection), Anica Mevissen, Louisa Katharina Rahm, Philipp Olbrich, Soufian Boulghoudan

**Affiliations:** 1 Cranio-and-Maxillo Facial Surgery, University Hospital Düsseldorf, Moorenstraße 5, 40225 Düsseldorf, Germany; 2 Heinrich-Heine-Universität Düsseldorf, Universitätsstrasse 1, 40225 Düsseldorf, Germany; 3 Orthopedics and Trauma Surgery, University Hospital Düsseldorf, Moorenstraße 5, 40225 Düsseldorf, Germany; 4 Dubai Health, Dubai, United Arab Emirates

**Keywords:** Postoperative delirium, Maxillofacial surgery, Geriatric assessment, Clock drawing test, Prospective, Medical research, Clinical trial design

## Abstract

Identifying high-risk patients for developing postoperative delirium (POD) is essential for optimizing the medical field’s human and financial resources through specialty-relevant geriatric assessments that can aid in establishing prehabilitation strategies. This study aims to identify geriatric screening tools to predict preoperative delirium and explore the high-risk elderly patients undergoing oral maxillofacial surgery. A comprehensive geriatric assessment encompassing 23 instruments was used to evaluate inpatients undergoing surgery under general anaesthesia, preoperatively and postoperatively. Selective intraoperative and postoperative variables were also assessed for their relation to POD occurrence. This prospective study included 90 patients (mean age 79.0 years) from August 2022 to August 2023. The POD rate in this cohort was (8.9% *n* = 8). The Clock-Drawing Test (CDT) was significantly associated with POD occurrence (*p* = 0.005). Significant associations were found between POD occurrence and operation type (*p* = 0.018), duration (*p* = 0.026), length of stay ( *p* = 0.002), and postoperative hemoglobin levels (*p* = 0.027). This study highlights the importance of comprehensive geriatric assessments in predicting POD in elderly patients. Future research should build on these findings to enhance preoperative care strategies and improve outcomes.

*Trial registration*: German Register of Clinical Studies, DRKSID DRKS00028614.

**Supplementary Information:**

The online version contains supplementary material available at 10.1038/s41598-024-78940-z.

## Background

Advancements in medicine allow for complex procedures in advanced-age patients who are frail and vulnerable. Chronological age alone cannot effectively gauge frailty. Stressors affect a frail body differently, and recovery rates vary significantly^1^. Identifying frail and non-frail older adults in the preoperative phase can optimize patient care plans. Frail older adults face higher risks of readmission, longer hospital stays, malnutrition, functional and cognitive decline, higher complication rates, new disabilities, and increased mortality^2,3^. Postoperative delirium (POD), which is a serious neuropsychiatric disorder associated with medical, cognitive, and functional impairment, is a common complication in this population^4^. A meta-analysis by Persico et al. found a significant association between delirium and frailty, with frail patients having a 2.2 times higher risk of developing delirium^5^. Mortality was found to increase significantly by 11% for every 48 h of delirium in patients aged 65 and older^6^.

Validated geriatric assessments are crucial for evaluating various aspects of elderly life, identifying patients at risk, and assessing functional abilities^7^. Standard preoperative geriatric assessments in oral and maxillofacial surgery are not well-established. Various validated geriatric assessments and screening tools are available in the literature, making it challenging to find a simple, goal-oriented collection of clinically significant assessments for daily preoperative use. Identifying assessments significantly associated with outcomes such as POD is essential for guiding effective prehabilitation plans. In view of the multifactorial nature of POD, it is important to support the implementation of non-pharmacological preventive intervention approaches.

Although 30–40% of delirium cases are considered preventable^8^, up to 72% of delirium events are not recognized or are misdiagnosed^9^. This could be due to the lack of awareness, variation in delirium presentation, its fluctuating nature, and difficulty in assessing cognitively impaired patients. Clinicians often rely on general observation rather than structured assessments, leading to frequent misdiagnosis^10,11^.

Oral and maxillofacial surgery add unique challenges compared to other surgical specialties. Postoperative communication and the detection of incoherent thinking can be limited due to intraoral and facial swelling, acute oral pain, restricted mouth movement, and tracheostomy, complicating the use of standard delirium assessment tools that rely on verbal communication. Thus, postoperative screening solely without baseline preoperative assessment might be insufficient.

This prospective cohort study aims to identify geriatric screening tools that can aid in predicting preoperative delirium and explore the high-risk group of elderly patients undergoing various oral maxillofacial surgical procedures.

## Methodology

### Study design

This prospective observational cohort study was conducted from August 2022 through August 2023 at the department of Oral, Maxillofacial, and Plastic Facial Surgery at Duesseldorf University Hospital in Germany. The study protocol was approved by the Ethics Committee of Heinrich Heine University in Germany (approval number: 2022 − 1810) and was conducted following the Declaration of Helsinki. The study is reported according to the criteria in the Strengthening the Reporting of Observational Studies in Epidemiology (STROBE) checklist.

### Participants

Patients were included if they were above 70 years old, planned for elective or emergency maxillofacial surgical procedures as inpatients in general anesthesia, and agreed to participate in the assessment pre- and postoperatively. Patients were excluded if they had severe dementia, were unable to participate in the assessment pre- or postoperatively, if the operation was planned as an outpatient procedure or was canceled, had Incomplete information that couldn’t be statistically evaluated, and were unwilling to cooperate with the research.

### Variables and study setting

The researchers performed a preoperative assessment following the study protocol. Table 1 presents the screening tools used in the study. During the preoperative period, several screening tools were used to assess different variables, including patients’ functional abilities, cognition, nutritional status, mobility and strength, emotions, hearing impairment, sleep disruptions, comorbidities, and delirium risk status.

POD was screened daily in the wards for patients enrolled in the study using three delirium screening tools. The intensive care unit (ICU) team assessed POD in the ICU. The POD evaluation period was seven days postoperatively or until the patient was discharged. All patients’ medical records were reviewed to collect patient demographics and relevant data from the anesthesia protocol, laboratory tests, and operation records.

### Statistical analysis

The collected data were recorded using Excel. Statistical analyses were performed with (version 2.2, The Jamovi Project, 2021 and Version 4.0, R Core Team, 2021) statistics programs. Statistical significance was set to a p-value of < 0.05 in all analyses. Exploratory data analysis and descriptive statistics were utilized for descriptive evaluation and to investigate the characteristics of the data. The Chi-square test and independent samples t-test were used to determine the association between different variables and the delirium rate in our dataset. For screening tools assessed at different time points, the Wilcoxon Signed-Rank Test was used. Binomial logistic regression was employed to evaluate the relationship between the presence or absence of POD and other independent variables. Other statistical tests, such as Welch’s t-test, were used when comparing two groups with unequal variances, and the Mann-Whitney U Test was utilized for comparing independent groups with non-normally distributed variables.

**Table 1:** Study protocol: comprehensive geriatric assessment in the preoperative and postoperative phases.

	Assessment Category	Assessment Tool	Reference
Preoperative Phase	Comorbidity status	The comorbidity-polypharmacy score (CPS)	^24^
		American society of Anesthesiologists score (ASA)	^25^
	Delirium risk status	Delirium risk assessment tool (DRAT)	^26^
		Anticholinergic Burden score (ACB)	^27^
	Function status	Katz-index of independence in activities of daily living	^28^
		Lawton-Brody instrumental activities of daily living scale (IADL)	^29^
		SARC-F (simple questionnaire to rapidly diagnose sarcopenia)	^30^
		Clinical frailty scale (CSF)	^31^
	Cognitive status	Six-items screener	^32^
		Clock drawing test (CDT)	^33^
	Nutritional status	Mini-Nutritional Assessment – short form (MNA-SF)	^34^
		Skinfold thickness over the triceps muscle	
		Body mass index (BMI)	
	Mobility and strength status	De Morton mobility index (DEMMI)	
			^35^
		Hand grip strength (hydraulic hand dynamometer)	
	Emotional status	World health organization Five-well-being index (WHO-5)	^36^
	Hearing impairment	Hearing handicap inventory for elderly – short form (HHIE-SF)	^37^
	Sleeping disruption	The Pittsburgh sleep quality index (PSQI)	^38^
		STOP-Bang score	^39^
		Insomnia severity Index (ISI)	^40^
Postoperative Phase	Delirium	Confusion assessment method (CAM)	^11^
		Nursing delirium screening scale (NuDESC)	^41^
		4AT delirium assessment tool	^42^
	Functional status	De Morton mobility index (DEMMI)	^35^
		Katz-index of independence in activities of daily living	^28^

## Results

The present cohort consists of 90 patients who underwent a Maxillofacial surgical procedure under general anesthesia during the data collection period and agreed to participate in this prospective study. Data from 43 women and 47 men, with an average age of 79.0 ± 5.7 years, were analyzed. An overview of demographic data is presented in the following Table (Table 2):

**Table 2:** Patient demographic and clinical characteristics.

Gender	Male	Female
*n* = 90	52.2% (*n* = 47)	47.8% (*n* = 43)
Age	78.0 ± 5.85	79.0 ± 5.43
BMI	25.7 ± 2.98	24.5 ± 5.07
ASA	ASA I	12.22% (*n* = 11)
	ASA II	38.89% (*n* = 35)
	ASA III	46.67% (*n* = 42)
	ASA IV	2.22% (*n* = 2)
Medical History	Hypertension	62.1% (*n* = 54)
	Diabetes	9% (*n* = 8)
	Cardiac disorders	46.7% (*n* = 42)
	Vascular diseases	62.2% (*n* = 56)
	Neurological disorders	27.7% (*n* = 25)
	Endocrine disorders	17.8% (*n* = 16)
	Psychiatric disorder	7.8% (*n* = 7)
	Oncological disorders	43.4% (*n* = 39)
Alcohol use disorder		23.9% (*n* = 11)
Smoking History	Never	46.2% (*n* = 41)
	Current	13.6% (*n* = 12)
	Ex-Smoker	37.5% (*n* = 33)
Medication	Analgesic opioids	8.9% (*n* = 8)
	Antidepressants	11.1% (*n* = 10)
	Parkinson’s medications	2.2% (*n* = 2)

Multiple screening tools and scores were evaluated during the preoperative assessment. These tools are categorized in Table 1 according to their assessment domains, which include comorbidity status, delirium risk status, functional status, cognitive status, nutritional status, mobility and strength status, emotional status, hearing impairment, and sleeping disruption. Their association with the occurrence of POD was assessed and statistically evaluated. The postoperative delirium rate of this cohort is 8.9% (*n* = 8). The summarized results of the association of comprehensive geriatric assessment instruments with POD are presented in Table 3.

**Table 3:** Comprehensive geriatric assessment instruments association with POD.

Screening Instruments		*n*	POD	Non-POD	*P*-Value
Comorbidity status					
ASA (*n* = 90)					*P* = 0.351
	ASA I	12.2% (*n* = 11)	9.1% (*n* = 1)	90.9% (*n* = 10)	
	ASA II	38.90% (*n* = 35)	2.9% (*n* = 1)	97.1% (*n* = 34)	
	ASA III	46.70% (*n* = 42)	14.3% (*n* = 6)	85.7% (*n* = 36)	
	ASA IV	2.2% (*n* = 2)	0	100% (*n* = 2)	
CPS (*n* = 90)					*p* = 0.371
	Mild	31.1% (*n* = 28)	3.6% (*n* = 1)	96.4% (*n* = 27)	
	Moderate	43.3% (*n* = 47)	14.9% (*n* = 7)	85.1% (*n* = 40)	
	Severe	14.5% (*n* = 13)	0	100% (*n* = 13)	
	Morbid	2.2% (*n* = 2)	0	100% (*n* = 2)	
Delirium risk status					
ACB score (*n* = 89)					*p* = 1.000
	Low score	86.5% (*n* = 77)	7.8%(*n* = 6)	92.2% (*n* = 71)	
	High score	13.5% (*n* = 12)	16.7% (*n* = 2)	83.3% (*n* = 10)	
DRAT (*n* = 90)					*p* = 0.480
	Low risk	51.1% (*n* = 46)	6.5% (*n* = 3)	93.5% (*n* = 43)	
	High risk	48.9% (*n* = 44)	11.4% (*n* = 5)	88.6% (*n* = 39)	
Functional status					
SARC-F (*n* = 90)					*p* = 0.126
	Low risk of sarcopenia	81.1% (*n* = 73)	6.8% (*n* = 5)	93.2% (*n* = 68)	
	High risk of sarcopenia	18.9% (*n* = 17)	17.6% (*n* = 3)	82.4% (*n* = 14)	
CFS (*n* = 74)					*p* = 0.083
	Non-Frail	81.1% (*n* = 60)	8.3% (*n* = 5)	91.7% (*n* = 55)	
	Frail	18.9% (*n* = 14)	21.4% (*n* = 3)	78.6% (*n* = 11)	
KATZ-Index, DEMMI score, IADL score are discussed below.					
Cognitive status					
Six-items-screener (*n* = 87)					*p* = 0.123
	No cognitive impairment	92.0% (*n* = 80)	7.5% (*n* = 6)	92.5% (*n* = 74)	
	Probable cognitive impairment	8.0% (*n* = 7)	28.6% (*n* = 2)	71.4% (*n* = 5)	
CDT (*n* = 89)					*p* = 0.005
	No signs of cognitive impairment	70.8% (*n* = 63)	7.9% (*n* = 5)	92.1% (*n* = 58)	
	probable cognitive impairment	22.5% (*n* = 20)	0	100% (*n* = 20)	
	Probable dementia	6.7% (*n* = 6)	50% (*n* = 3)	50% (*n* = 3)	
Nutritional status					
MNA- SF (*n* = 90)					*p* = 0.139
	Well-nourished	43.3% (*n* = 39)	2.5% (*n* = 1)	97.4% (*n* = 38)	
	Risk of malnutrition or Malnourished	56.7% (*n* = 51)	13.7 (*n* = 7)	86.3 (*n* = 44)	
Emotional status					
WHO-5 (*n* = 88)					*P* = 0.799
	reduced level of well-being	14.4%(*n* = 13)	15.38% (*n* = 2)	84.6% (*n* = 11)	
	satisfactory or good level of well-being	83.3% (*n* = 75)	8% (*n* = 6)	92% (*n* = 69)	
Hearing impairment					
HHIE-S (*n* = 89)					*p* = 1.000
	No hearing impairment	80.9% (*n* = 72)	9.7% (*n* = 7)	90.3% (*n* = 65)	
	Hearing impairment	19.1% (*n* = 17)	5.9% (*n* = 1)	94.1% (*n* = 16)	
Sleeping disruption					
PSQI (*n* = 88)					*p* = 0.142
	Healthy sleepers	67%(*n* = 59)	13.6% (*n* = 8)	86.4% (*n* = 51)	
	Poor sleepers	23.9%(*n* = 21)	0	100% (*n* = 21)	
	Chronic sleep disorders	9.1% (*n* = 8)	0	100% (*n* = 8)	
ISI (*n* = 88)					*p* = 0.211
	No clinically significant insomnia	73.9% (*n* = 65)	12.3% (*n* = 8)	87.7% (*n* = 49)	
	Subthreshold insomnia	19.3%(*n* = 17)	0	100% (*n* = 17)	
	Clinical insomnia	6.8%(*n* = 6)	0	100% (*n* = 6)	
STOP-BANG (*n* = 90)					*p* = 0.364
	Low risk for OSA	28.9% (*n* = 26)	7.7% (*n* = 2)	92.3% (*n* = 24)	
	Moderate risk for OSA	60.0% (*n* = 54)	9.3% (*n* = 5)	90.7% (*n* = 49)	
	High risk for OSA	11.1% (*n* = 10)	10.0% (*n* = 1)	90% (*n* = 9)	

The Comorbidity-Polypharmacy Score (CPS) quantitatively measures the comorbidity’s severity and can be used as an initial assessment. In this study, POD did not occur in the two highest-risk CPS categories, and the statistical association between POD occurrence and CPS risk level was not significant, with χ²(3) = 4.55, *p* = 0.371, and Cramer’s V = 0.225. The risk of delirium was assessed using the DRAT and the ACB score. Although higher rates of POD were observed in participants with elevated scores, neither tool demonstrated a significant association between POD occurrence and higher delirium risk categories (χ²(1) = 0.651, *p* = 0.480, Cramer’s V = 0.085 for DRAT; χ²(1) = 0.0566, *p* = 1.000, Cramer’s V = 0.0252 for ACB). Additionally, evaluations of frailty and sarcopenia risk using the SARC-F and CFS also showed no significant statistical relationship with POD occurrence.

As part of the comprehensive geriatric assessment, the functional status and basic activities of daily living were assessed using the Katz Index and IADL score. The mobility status was assessed and documented with DEMMI score. The Katz Index shows average values of 6 ± 1.1 (*n* = 90) at admission and 6 ± 1.14 (*n* = 83) at discharge. The comparison of both values showed no significant difference z = 10.0, *p* = 0.072, *r* = 0.224. For the IADL score, the values at admission were 8.0 ± 2.04 for women and 5.00 ± 1.30 for men. The analysis of the DEMMI reveals values of 92.5 ± 27.4 (*n* = 88) at admission and 74 ± 26.8 (*n* = 81) at discharge. Comparing the values at admission and discharge shows a significant decline in the DEMMI values (z = 29.0, *p* = 0.021, *r* = 0.265) (Supplemental Fig. 1).

A binomial logistic regression was performed to demonstrate the effect of potential mobility impairment on the POD rate. The binomial logistic regression model is significant with χ²(5) = 15.0, *p* = 0.010, and Nagelkerke’s R² = 0.419. The model’s accuracy is 93.6%, with a specificity of 98.6% and a sensitivity of 33.3%. Among the variables examined, the preoperative DEMMI (*p* = 0.006) and the DEMMI at discharge (*p* = 0.007) are significant. With an OR = 1.176 (95% CI [1.047, 1.322]), the DEMMI at admission is a positive predictor, and with an OR = 0.813 (95% CI [0.701, 0.944]), the DEMMI at discharge is a negative predictor. All model coefficients and odds are listed in the following table (Supplemental Table 1).

The MNA-SF score assessment tool was utilized to assess the preoperative nutritional status. Although a higher rate of POD was observed in the group of patients at risk of malnutrition and those who were malnourished, a significant relationship could not be established, with χ²(2) = 4.69, *p* = 0.139, and Cramer’s V = 0.228. To further assess nutritional status, skinfold thickness measurements over the triceps muscle were performed. The results indicated that women had a mean skinfold thickness of 12.0 ± 7.21 mm, while men had a mean value of 14.0 ± 6.49 mm. Additionally, grip strength testing was conducted, revealing mean values of 22.0 ± 6.50 kg for women and 34.0 ± 10.6 kg for men.

Two tools were used to assess cognitive function. The six-item screener did not show a statistically significant result (χ²(1) = 3.42, *p* = 0.123, Cramer’s V = 0.195). In contrast, with the clock-drawing test, a significant relationship was evident (χ²(1) = 14.4, *p* = 0.005, Cramer’s V = 0.402), indicating that CDT scores are moderately predictive of delirium occurrence. Different screening tools were used to assess sleep disorders; none of the tests revealed a significant relationship to POD occurrence. Only patients with an increased risk of obstructive sleep apnea showed a higher rate of POD occurrence.

In this study, patients underwent a wide range of surgeries that differed in complexity, duration, and stress levels. When comparing various types of surgeries, tumor patients, as the most vulnerable group (major surgery), exhibited a delirium rate of 31.6% (*n* = 6) (Supplemental Table 2). The Chi-Square test revealed a statistically significant association between operation type and POD occurrence (χ²(7) = 16.94, *p* = 0.018, Cramer’s V = 0.434). The patients who underwent major surgery (*n* = 19) were analyzed separately to examine the relationship between POD occurrence and all the screening tools and scores used; none showed a significant relationship.

A statistically significant difference was observed in the operation duration between patients with and without delirium. The operation duration for patients without delirium was 229 min shorter (95% CI [36, 422]), t(7.34) = 2.78, *p* = 0.026, Cohen’s d = 1.26 (Welch’s t-Test). A Mann-Whitney U test was conducted to determine if there was a difference in the length of hospital stay between patients with and without delirium. The distributions of the two groups differed significantly, Kolmogorov-Smirnov *p* < 0.001. There was a significant difference in the length of hospital stay in days between patients with and without delirium, (U = 105, *p* = 0.002, *r* = 0.677).

As hemoglobin (Hb) level is considered one of the risk factors for delirium, we examined it in the pre- and postoperative phases. The preoperative Hb levels had no significant relationship with POD as indicated by t(84.0)=-0.910, *p* = 0.365, Cohen’s d=-0.338. However, the difference in Hb levels in the postoperative phase was significant, with patients experiencing POD having Hb levels 1.881 mg/dL (95%-CI[3.55, 0.215]) lower than those without POD, shown by t(75.0)=-2.249, *p* = 0.027, Cohen’s d=-0.892.

Postoperative ICU stay and the presence of a tracheostomy are known risk factors for POD. The analysis revealed higher rates of POD in patients with a postoperative ICU stay and those with a tracheostomy. However, these results were not statistically significant. For tracheostomy cases, delirium occurred in 20% of cases compared to 10% in non-tracheostomy cases(χ²(1) = 0.172, *P* = 0.678, Cramer’s V = 0.044). For ICU stay cases, delirium occurred in 33.33% of cases compared to 10.26% in non-ICU cases, also showing no significant association in this cohort (χ²(1) = 3.004, *P* = 0.083, Cramer’s V = 0.183).

In this study, three different delirium screening tools, 4AT, NuDESC, and CAM, were used in the postoperative phase. The results showed no discrepancies, as each tool consistently indicated either the presence or absence of POD. This uniformity highlights the reliability and potential interchangeability of these screening methods in detecting delirium within the studied cohort.

## Discussion

### Importance and challenges of comprehensive geriatric assessment

Comprehensive geriatric assessment is necessary in light of the increasing elderly population and the complexity of surgeries performed. The current treatment modalities for patients with multiple comorbidities are fragmented and poorly coordinated. Holistic, patient-centered management approaches, which consider all factors influencing the patient’s condition and treatment outcome, are recommended^12^. Despite its importance, there is no consensus on geriatric assessment in surgical specialties^13^. Each specialty has unique characteristics and postoperative limitations. Integrating a full geriatric assessment into the daily routine of a busy surgical team is challenging, highlighting the need for objective, goal-oriented instruments.

A noteworthy approach was implemented in the EASE (*Elder-Friendly Approaches to the Surgical Environment*) initiative to create an evidence-based, elder-friendly surgical environment by incorporating geriatric assessments for patients undergoing emergency surgeries in general surgery departments. This resulted in many positive outcomes, including a 19% reduction in mortality (51 of 153 [33.3%] vs. 19 of 140 [13.6%]; *P* < 0.001), as well as reduced complications, length of stay, and discharge to care facilities^14^. These positive results are very promising and encouraging, suggesting that similar practices should be adopted in elective settings.

### POD in OMFS

POD is a serious condition that delays recovery, leads to complications, and increases hospital length of stay. Early recognition and management are essential to reduce these adverse outcomes, particularly in patients undergoing major surgical procedures. The incidence rate of POD in our cohort was 8.9%, rising to 31.6% in patients undergoing major surgery, consistent with previously published data^15,16^. Major operations with extended durations are known risk factors for POD in OMFS. Previous studies reported an increased risk with operation durations of 6–10 h^17,18^, and one study noted that each additional 10 min of surgery increases the odds of POD by 3.2%^19^. Kinoshita et al. noted that POD could lead to OMFS-relevant complications such as flap necrosis^20^, which not only increases the length of stay but also increases exposure to multiple surgical procedures under general anesthesia. This may lead to further complications and delay additional adjunct treatment in patients requiring chemotherapy and radiotherapy.

Training medical teams to recognize and manage POD, particularly the hypoactive type, is crucial. Some studies have discussed that the presence of a tracheostomy in the postoperative period can increase the possibility of overlooking or misdiagnosing hypoactive delirium due to challenged communication^19,21^. In our cohort, this could not be elaborately reported due to the limited number of patients with tracheostomies. Future studies focusing on this subgroup may help identify the proper management.

Previous prospective studies related to POD failed to report a cognitive baseline assessment. In our study, the CDT was a significant predictor of POD, underscoring its utility in cognitive preoperative evaluations. This finding aligns with previous research by Goldstein et al., which found a strong association between cognitive impairment and postoperative delirium^15^. Routine use of cognitive screening tools such as the CDT in preoperative assessments could enhance the early identification of at-risk patients.

The association between the change in hemoglobin level post-surgery in patients who developed POD aligns with findings by Makiguchi et al., suggesting that monitoring and managing hemoglobin levels could be crucial in preventing POD^22^.

Given the association between obstructive sleep apnea (OSA) and POD and the possibility of modifying or early intervention in some cases^23^, incorporating OSA screening into preoperative assessments using an instrument like STOP-BANG could be beneficial, as higher-risk groups showed an elevated POD rate in our cohort. Although Insomnia has been reported as a significant risk factor in the OMFS literature^22^, this association couldn’t be established in our cohort using the Insomnia Severity Index.

### Comprehensive and individualized approaches

A comprehensive approach that sets individualized goals for each patient is essential. Proper interventions in terms of the choice of surgical procedure and monitoring intraoperative factors such as operation duration should be applied^12^. Efficiently directing resources to high-risk groups identified through comprehensive geriatric assessments could improve patient outcomes and reduce healthcare costs. Future research should continue refining these strategies and exploring additional factors contributing to POD.

### Study strengths and limitations

Our cohort study is the first, to our knowledge, to examine a comprehensive geriatric assessment, including the multifactorial aspects of POD, in the field of oral and maxillofacial surgery. Besides being prospective, the strengths of our study include using multiple instruments in parallel to determine which is most clinically relevant, time-efficient, and practical for application without extensive training. Another strength that many previous studies have lacked is the inclusion of baseline cognitive assessments, which are highly relevant to delirium diagnosis.

Despite the relatively adequate number of patients included, we acknowledge that the sample size is still insufficient. One of the main difficulties encountered during the recruitment phase was that patients felt overwhelmed by the number of examinations required, in addition to ongoing medical diagnostics and examinations. Integrating the most relevant tests that carry significant clinical outcomes into routine examinations during all preoperative visits could easily address this issue.

Another limitation of our study is the low number of delirium cases diagnosed compared to the many variables tested, which introduces a risk of over-fitting. Future studies should focus on comprehensive but goal-specific instruments that objectively document clinical findings and can be reproduced by each member of the team. It is also crucial to utilize resources by focusing on high-risk groups, particularly those undergoing longer and major operations.

## Conclusions

**Fig. 1:**
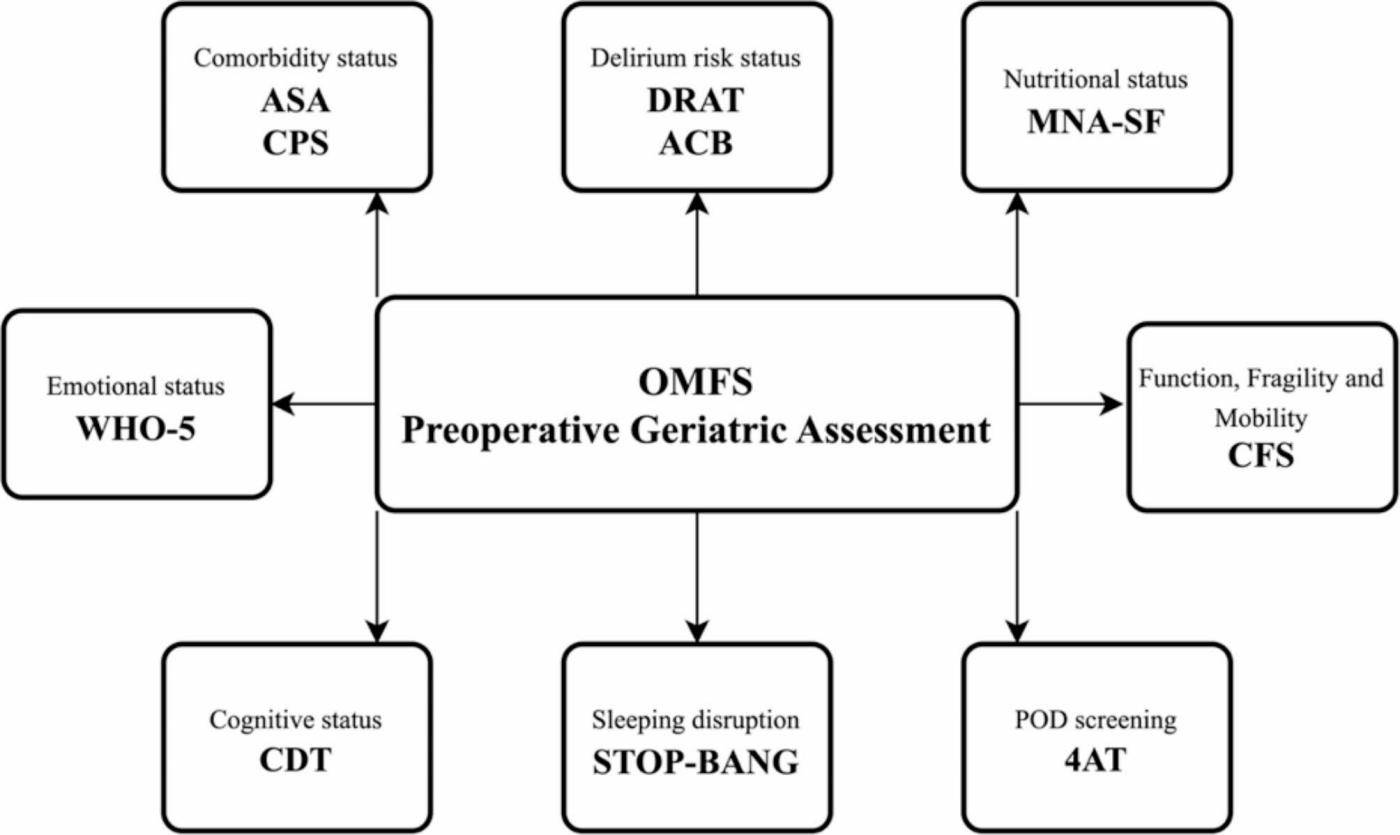
OMFS preoperative geriatric assessment for postoperative delirium.

Our findings identified multiple geriatric assessment instruments relevant to OMFS that can be easily assessed in the preoperative phase. A suggested comprehensive assessment model, which meets the diversity of POD factors and includes the tests that showed clinical relevance to POD, is presented in Fig. 1. POD has multiple predisposing factors that are not always modifiable and many precipitating factors that could be detected and prevented. Identifying high-risk groups and educating them and their families is key to a prehabilitation plan. Additionally, educating and training the medical team about the seriousness of POD development and its short- and long-term complications is crucial. Focusing on early detection through regular screening, applying non-pharmacological measures, and using pharmacological interventions when necessary are the first steps to developing a specialty-specific package of measures that can significantly improve patient care and outcomes.

## Electronic supplementary material

Below is the link to the electronic supplementary material.

Supplementary Material 1

Supplementary Material 2

## Data Availability

The datasets used and/or analysed during the current study are available from the corresponding author on reasonable request.
