# Supplementary material for: Comprehensive geriatric assessment for predicting postoperative delirium in oral and maxillofacial surgery: a prospective cohort study
Source: Sci Rep. 2024 Nov 11;14:27554. doi: 10.1038/s41598-024-78940-z (PMC11554771; doi:10.1038/s41598-024-78940-z)
Supplement: Supplementary file 1 — Supplementary Material 1 [file 41598_2024_78940_MOESM1_ESM.docx]

**Tables legends (Supplemental)**

**Additional Table 1**: Model Coefficients and Odds for IADL, and Comparison of Admission and Discharge Scores for DEMMI and Katz-Index

| Model Fit Measures | | | | | | | | | | | | | | | | | | | | | | | | | | | | | |  |  |  |  |  |  |
| --- | --- | --- | --- | --- | --- | --- | --- | --- | --- | --- | --- | --- | --- | --- | --- | --- | --- | --- | --- | --- | --- | --- | --- | --- | --- | --- | --- | --- | --- | --- | --- | --- | --- | --- | --- |
|  | | | | | | | | | | | | | | | | | | Overall Model Test | | | | | | | | | | | |  |  |  |  |  |  |
| Model | | | Deviance | | | | AIC | | | BIC | | | | R²McF | | R²N | | χ² | | | | df | | | p | | | | |  |  |  |  |  |  |
| 1 |  | | 27.3 | |  | | 39.3 | |  | 53.4 | | |  | 0.355 |  | 0.419 |  | 15.0 | | |  | 5 |  | | 0.010 | | |  | |  |  |  |  |  |  |
|  | | | | | | | | | | | | | | | | | | | | | | | | | | | | | |  |  |  |  |  |  |
| Model Coefficients – POD yes/no | | | | | | | | | | | | | | | | | | | | | | | | | | | | | | | | | | | |
|  | | | | | | | | 95% Confidence Interval | | | | | | | |  | | | | | | | | | | | | | | | | 95% Confidence Interval | | | |
| Predictor | | | | Estimate | | | | Lower | | | | Upper | | | | SE | | | | Z | | | | | | p | | | Odds ratio | | | Lower | | Upper | |
| Intercept | |  | | 1.05582 | |  | | -1.8323 | | |  | 3.9439 | | |  | 1.4735 | | |  | 0.71652 | | | |  | | 0.474 |  | | 2.874 | |  | 0.160 |  | 51.620 |  |
| DEMMI | |  | | 0.16252 | |  | | 0.0460 | | |  | 0.2791 | | |  | 0.0595 | | |  | 2.73258 | | | |  | | 0.006 |  | | 1.176 | |  | 1.047 |  | 1.322 |  |
| D: DEMMI | |  | | -0.20653 | |  | | -0.3558 | | |  | -0.0572 | | |  | 0.0762 | | |  | -2.71100 | | | |  | | 0.007 |  | | 0.813 | |  | 0.701 |  | 0.944 |  |
| Katz-Index | |  | | -24.21168 | |  | | -4856.6554 | | |  | 4808.2321 | | |  | 2465.5778 | | |  | -0.00982 | | | |  | | 0.992 |  | | 3.05e-11 | |  | 0.000 |  | Inf |  |
| D :Katz-Index | |  | | 23.94063 | |  | | -4808.5032 | | |  | 4856.3845 | | |  | 2465.5779 | | |  | 0.00971 | | | |  | | 0.992 |  | | 2.50e+10 | |  | 0.000 |  | Inf |  |
| IADL | |  | | 0.00400 | |  | | -0.6138 | | |  | 0.6218 | | |  | 0.3152 | | |  | 0.01270 | | | |  | | 0.990 |  | | 1.004 | |  | 0.541 |  | 1.862 |  |
| Note. Estimates represent the log odds of "POD yes/no = yes" vs. "POD yes/no = no" | | | | | | | | | | | | | | | | | | | | | | | | | | | | | | | | | | | |

**Additional Table 2** : Incidence of POD by Operation Type

|  |  |  | POD |  | POD rate |
| --- | --- | --- | --- | --- | --- |
| Operation Type | No. of Patients | Percentage | No | Yes |  |
| Dentoalveolar surgery | 15 | 16.7 % | 15 | 0 |  |
| Biopsy/ Panendoscopy | 9 | 10.0 % | 9 | 0 |  |
| Soft tissue reconstruction | 8 | 8.9 % | 8 | 0 |  |
| Osteonecrosis related surgery | 15 | 16.7 % | 15 | 0 |  |
| Trauma | 13 | 14.4 % | 12 | 1 | 7.7% |
| TMJ related surgery | 2 | 2.2 % | 2 | 0 |  |
| Major tumor surgery | 19 | 21.1 % | 13 | 6 | 31.6% |
| others | 9 | 10.0 % | 8 | 1 | 11.1% |
| Total | 90 |  | 82 | 8 |  |
