# Supplementary material for: Comprehensive geriatric assessment for predicting postoperative delirium in oral and maxillofacial surgery: a prospective cohort study
Source: Sci Rep. 2024 Nov 11;14:27554. doi: 10.1038/s41598-024-78940-z (PMC11554771; doi:10.1038/s41598-024-78940-z)

**Figures legends (Supplemental)**

**Additional Figure 1**: The difference between admission and discharge values in Katz-Index and DEMMI score. (* p <0,05; ** p <0,01; *** p <0,001).


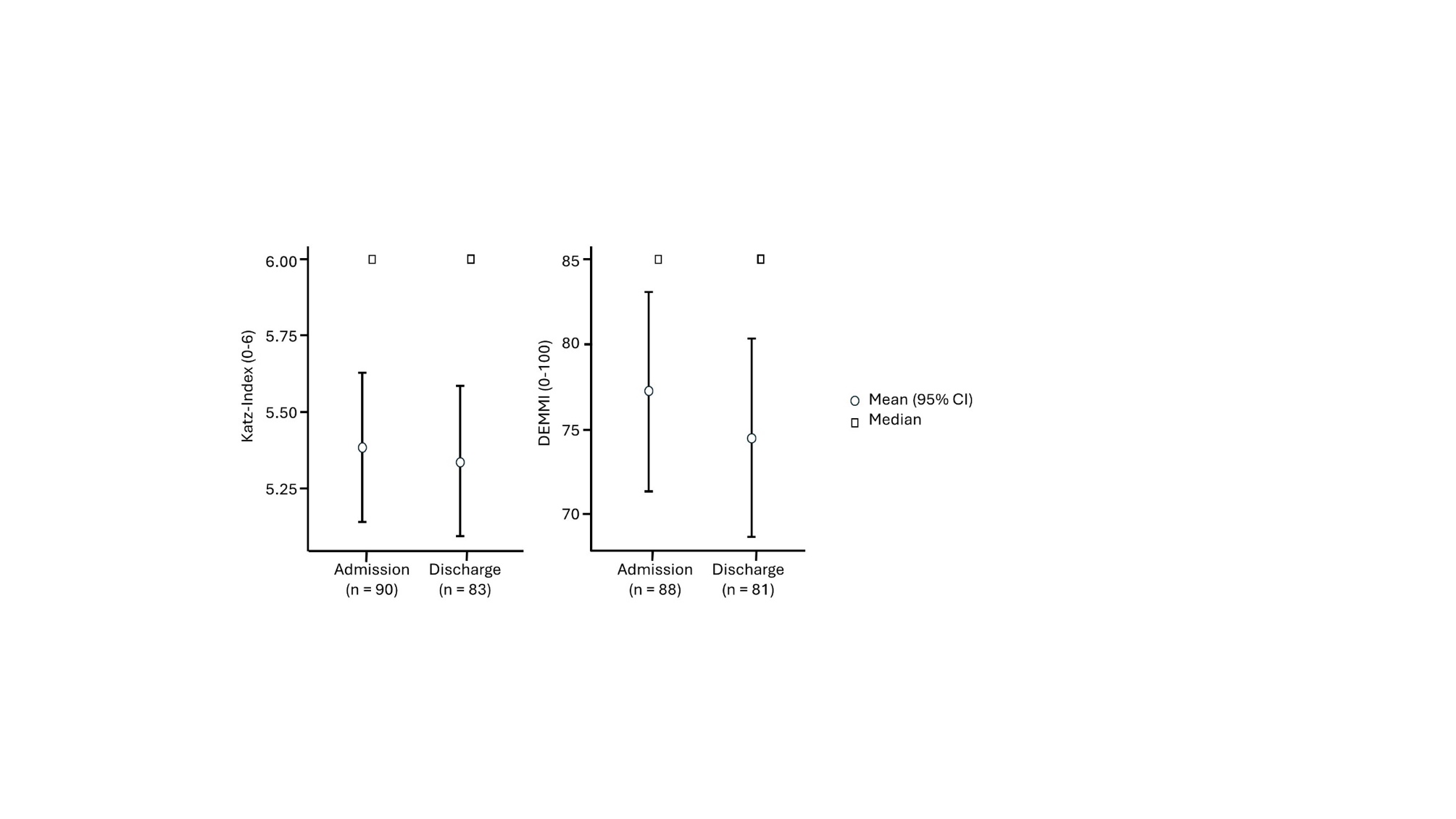


**Additional Figure 2** : Comparison of Surgery Duration based on POD occurrence (* p <0,05; ** p <0,01; *** p <0,001).


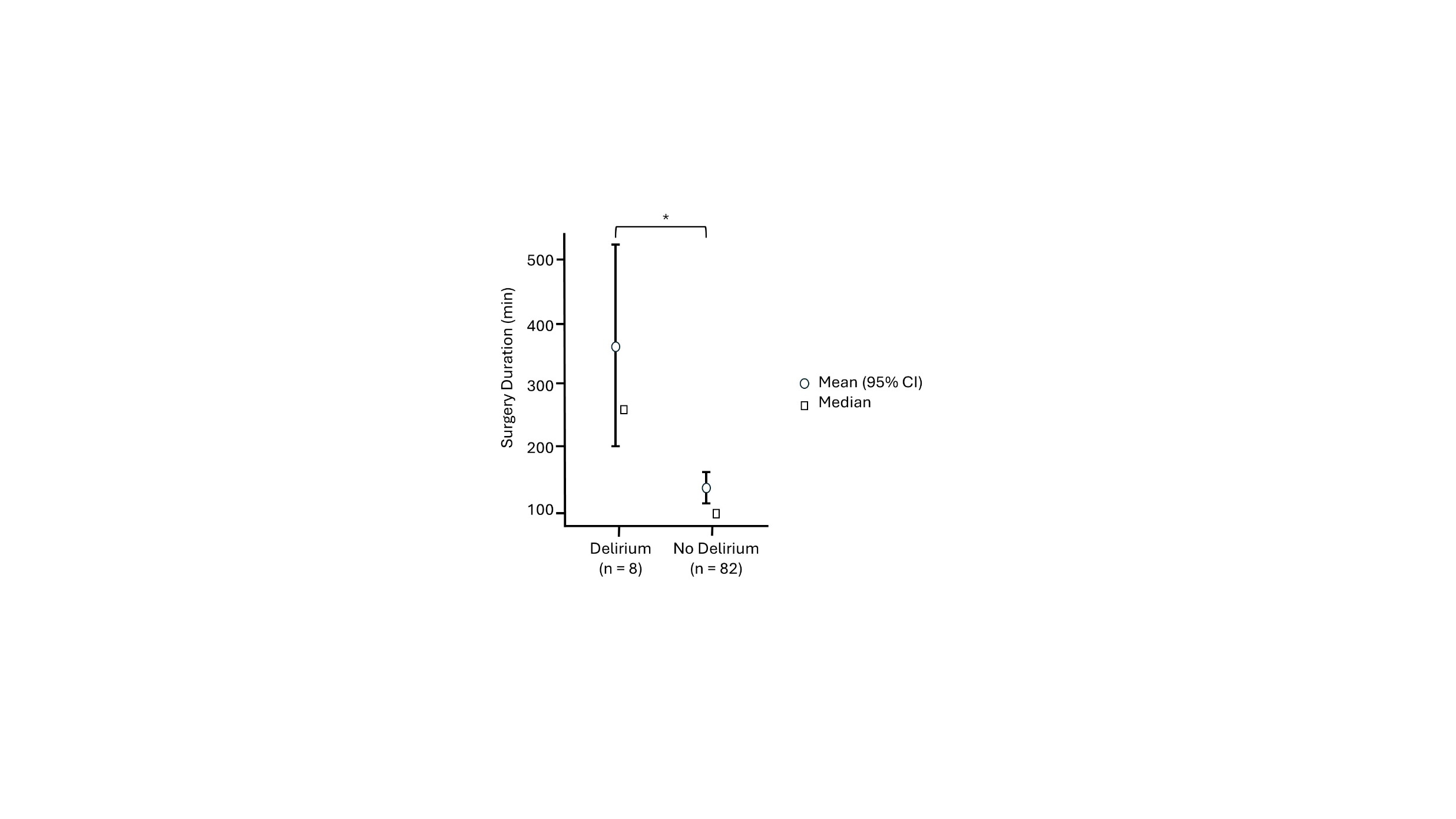


**Additional Figure 3**: Comparison of length of stay based on POD occurrence (* p <0,05; ** p <0,01; *** p <0,001).


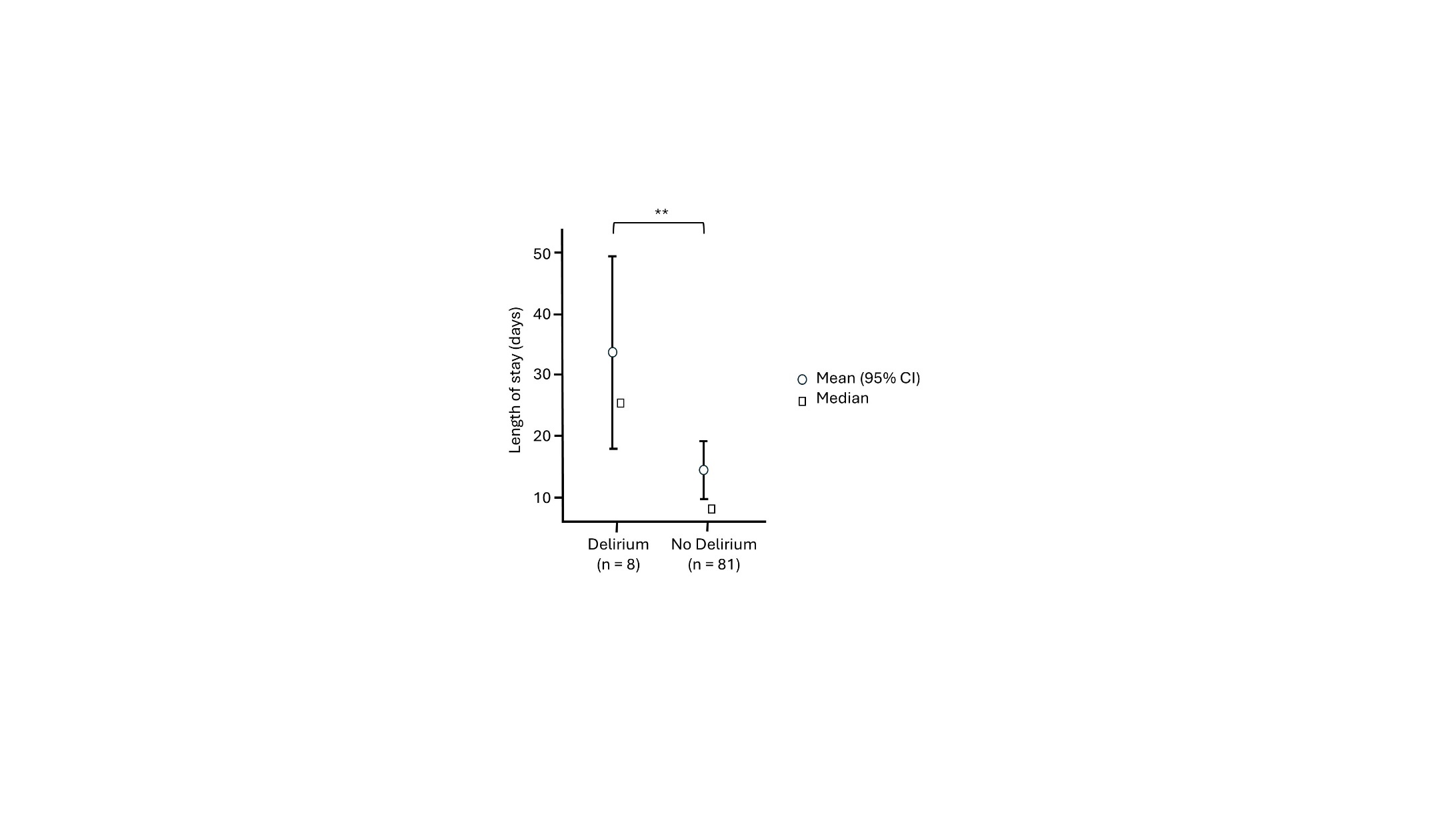


**Additional** **Figure 4** : Comparison of preoperative (left) and postoperative (right) Hb-level based on POD occurrence (* p <0,05; ** p <0,01; *** p <0,001).


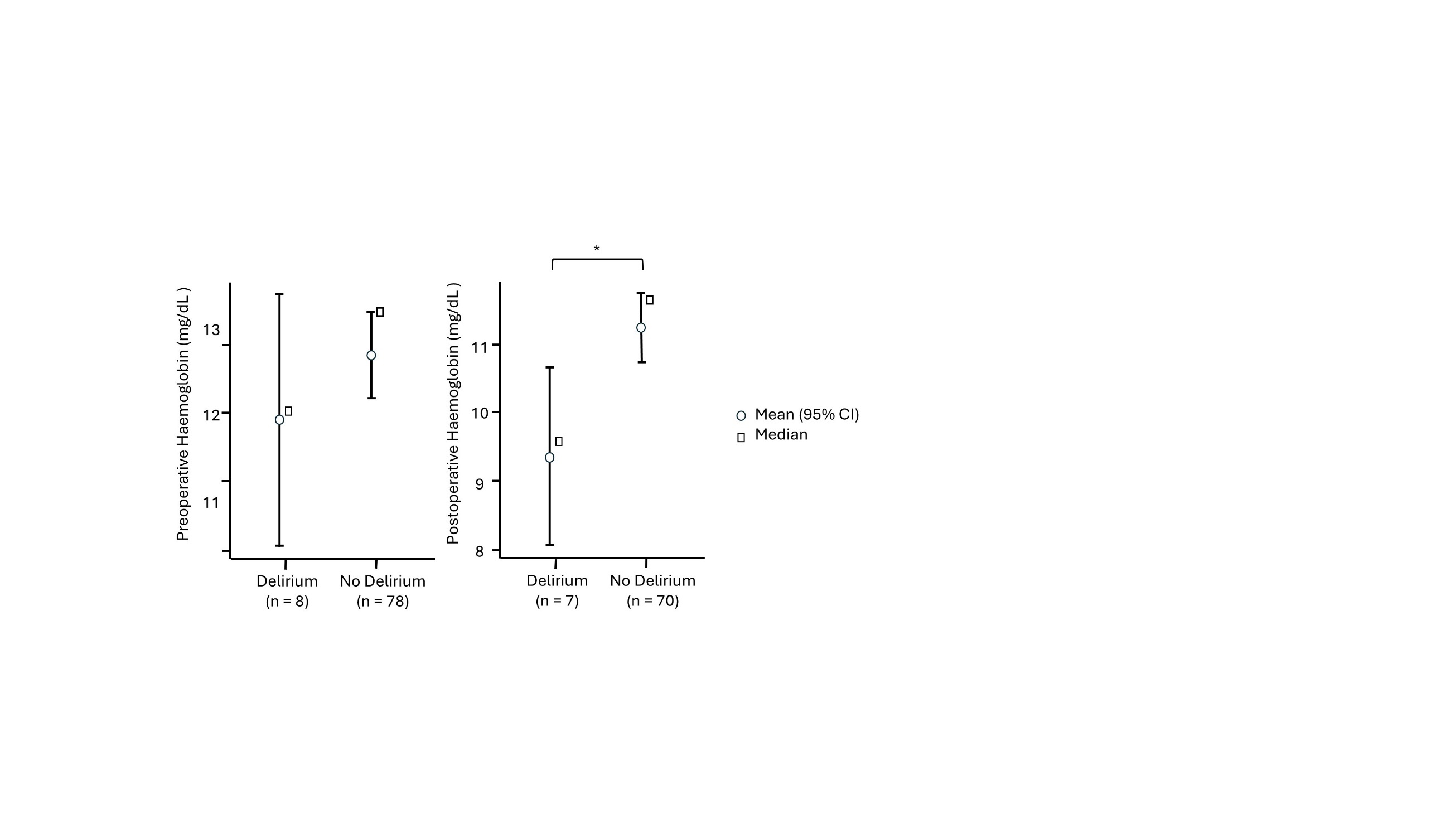

Supplement: Supplementary file 2 — Supplementary Material 2 [file 41598_2024_78940_MOESM2_ESM.docx]
